# Elevated CO_2_ Enhanced the Antioxidant Activity and Downregulated Cell Wall Metabolism of Wolfberry (*Lycium barbarum* L.)

**DOI:** 10.3390/antiox11010016

**Published:** 2021-12-22

**Authors:** Ze Liang, Zisheng Luo, Wenxuan Li, Mingyi Yang, Lei Wang, Xingyu Lin, Li Li

**Affiliations:** 1 Key Laboratory of Agro-Products Postharvest Handling, Fuli Institute of Food Science, Ministry of Agriculture and Rural Affairs, College of Biosystems Engineering and Food Science, Zhejiang University, Hangzhou 310058, China; liangze0803@zju.edu.cn (Z.L.); luozisheng@zju.edu.cn (Z.L.); wenxuanli@zju.edu.cn (W.L.); ymy008@zju.edu.cn (M.Y.); wangley@zju.edu.cn (L.W.); xingyu@zju.edu.cn (X.L.); 2 National-Local Joint Engineering Laboratory of Intelligent Food Technology and Equipment, Zhejiang University, Hangzhou 310058, China; 3 Zhejiang Key Laboratory for Agro-Food Processing, Zhejiang University, Hangzhou 310058, China; 4 Zhejiang Engineering Laboratory of Food Technology and Equipment, Zhejiang University, Hangzhou 310058, China; 5 Ningbo Research Institute, Zhejiang University, Ningbo 315100, China

**Keywords:** MAP, CO_2_ atmosphere, cell wall metabolism, antioxidant activity, wolfberry

## Abstract

Modified atmosphere packaging (MAP) has been widely known to delay the postharvest fruit senescence; nevertheless, its effect on antioxidant activity and cell wall metabolism of wolfberry fruit is largely unknown. The present study investigated the impact of elevated CO_2_ on the quality attributes and cell wall degradation of wolfberry fruit during storage. The results showed that 10% CO_2_ better maintained the physiological quality and conferred the reduction in weight loss, decay index, and color change. Higher 2,2′-azino-bis (3-ethylbenzothiazoline-6-sulfonic acid) (ABTS) and 1,1-diphenyl-1-picrylhydrazil (DPPH) radical scavenging activity, total phenol and flavonoid content, and superoxide dismutase (SOD) and catalase (CAT) activity of wolfberry were detected at elevated CO_2_ concentrations. Elevated CO_2_ atmosphere contributed to the maintenance of the cell integrity, the decrease of cell wall degradation (polygalacturonase, pectate lyase, cellulase, and β-glucosidase), and the increase of cellulose and proto pectin content. Overall, we revealed the potential mechanism of elevated CO_2_ on the antioxidant activity enhancement and cell wall homeostasis of fresh berry fruit.

## 1. Introduction

Wolfberry (*Lycium barbarum* L.), a species of the Solanaceae family, is a woody and perennial plant and mainly grown in Asian countries [1]. It is traditionally used in Chinese, Korean, Japanese, and Vietnamese medicine [2]. In China, the total production of wolfberry is around 400 thousand tonnes, among which more than 80% is being cultivated in Ningxia, with Zhongning County as the center [3]. In 2019, the wolfberry planting area in Ningxia covered around 166.6 thousand acres, and the output reached up to 102 thousand tons. Due to its nutraceutical content and higher consumption of fresh and dried berries, the planting area of wolfberry in China is gradually increasing. As a result, it has become one of the essential economic crops in the northwestern provinces of China. In addition to the Asian countries, wolfberry has grown in Canada, the USA, Romania, and Italy, where some new varieties have been cultivated [4]. Wolfberries are thought to provide a wide source of health benefits. They are rich in vitamins A, B_1_, B_2_, and C, as well as other essential nutrients for maintaining eye health. Polysaccharides in wolfberries provide anti-aging effects and strengthen kidney functions [5,6]. However, due to the influence of different ecological environments and cultivation techniques, the quality of wolfberry is uneven, and physiological disorders and quality deterioration problems such as fruit rotting and softening can occur during the postharvest storage of fresh wolfberry fruits. Thus, developing low-cost, efficient, and user-friendly postharvest preservation techniques is of current importance to preserve fresh wolfberry fruits’ freshness quality, thus increasing its availability and preventing economic and food loss.

In general, after the fresh wolfberry fruit is harvested, the inner water vapor pressure is lower than on the surface, resulting in transpiration and water loss. The fresh wolfberry fruit, after harvest, loses its photosynthetic capacity due to the decomposition of chlorophyll, and the increase in vitamins loss and anthocyanins decrease contribute to fruit color change [7]. When a large amount of active oxygen accumulates, the antioxidant metabolism of the tissue changes [8]. In the process of ripe fruit aging, the hardness of the fresh wolfberry fruit gradually decreases, and the structure of the cell wall also undergoes sudden changes with increasing membrane permeability [9]. Most studies showed that the degradation of cell wall components is caused by cell wall hydrolase, especially closely related to polygalacturonase and pectin methylesterase [10]. Understanding the changes in antioxidant activity and cell wall structure in post-harvested wolfberry fruits is of great significance in determining the possible mechanism of quality preservation in modified atmosphere packaging (MAP) storage conditions.

Fresh wolfberry fruit has a short shelf life and is perishable, mostly converted to its dried form. However, the drying process of fresh fruits will cause the loss of many nutrients and active constituents, restricting the quality of wolfberry products. MAP is an easy-to-operate and environmentally friendly method to preserve various fruits and vegetables [11]. It has been widely used by adjusting the gas composition and proportion in the storage environment to maintain the freshness and nutritional value and further extend the shelf life of fruits and vegetables, such as mandarin orange [12], strawberry [13], corns [14], and cherry tomatoes [15]. As such, studies have been conducted on using MAP for fruit preservation, such as increasing the total phenol content and antioxidant activity in blueberry [16], reducing respiration rate and ethylene synthesis of the products to maintain flesh firmness in cherry laurel fruit [17]. Therefore, MAP treatment might be a potential strategy for fruit quality control.

The present study intended to explore the quality changes of postharvest wolfberry fresh fruit, employing controlled atmosphere preservation. It also analyzed the role of the antioxidant system and cell wall structure in mitigating the fruit rotting and softening in fresh wolfberry fruit. This will help to understand the effect of different MAP conditions on fruit freshness quality and thus could assist in developing a better preservation condition for the commercial storage of fresh wolfberry fruit.

## 2. Materials and Methods

### 2.1. Wolfberry Materials and Treatments

The wolfberry (*Lycium barbarum* L. cv. Ningqi No. 10) fruits (with diameter of 8–20 mm) were obtained from the local producer (Zhongning, Ningxia province, China) and then transported to the laboratory in a close plastic container (295 × 230 × 185 mm, Lock&lock, Suzhou, China). Fruits with uniform ripeness, color, and size were selected as experimental samples. They were randomly divided into four groups exposed to CT (air), 5% CO_2_ + 5% O_2_, 10% CO_2_ + 5% O_2_, and 15% CO_2_ + 5% O_2_ atmosphere and were checked by catharometer (MOCON Europe A/S, Ringsted, Denmark). Samples were stored at 0 ± 0.5 °C for 28 d, and their quality was investigated for every 7 d (D0, D7, D14, D21, and D28). In addition, fruits samples were immediately frozen in liquid nitrogen and stored in an ultra-low temperature freezer (−80 °C). We used a grinder (MM-400, Retsch, Haan, Germany) to grind frozen samples into powder for further analysis.

### 2.2. Fruit Quality Traits

#### 2.2.1. Weight Loss Rate

The weight loss rate was calculated by the decreased weight at each sampling point to its initial weight before storage. Three replications were performed. The weight loss was determined using the following Equation (1).

(1) $$\mathrm{Weight}\;\mathrm{loss}\;\mathrm{rate}\left(\%\right)=\frac{\mathrm{initial}\;\mathrm{weight}-\mathrm{sampling}\;\mathrm{time}\;\mathrm{weight}}{\mathrm{initial}\;\mathrm{weight}}$$

#### 2.2.2. Rotten Index

The fruits were examined at four rotten levels according to the rotted area of the fruit surface, namely, I, 0% ≤ rotted area ≤ 25%; II, 25% < rotted area ≤ 50%; III, 50% < rotted area ≤ 75%; IV, 75% < rotted area ≤ 100%. Three replications were performed. The rotten index was calculated by using the following Equation (2).

(2) $$\mathrm{Rotten}\;\mathrm{index}\;\left(\%\right)=\frac{\Sigma \;\left(\mathrm{fruit}\;\mathrm{quality}\times \mathrm{corresponding}\;\mathrm{rot}\;\mathrm{level}\right)}{4\times \mathrm{overall}\;\mathrm{fruit}\;\mathrm{quantity}}$$

#### 2.2.3. Color

The color value of fruits was measured according to the method mentioned by Li et al. [18]. Five wolfberries were taken randomly from each group and tested two times around the equatorial region. The colorimeter (CR-400, KONICA MINOLTA, Tokyo, Japan) recorded the wolfberry’s L*, a*, and b* values. The color attributes were determined by color coordinates of L* (L* = 0 (black) and L* = 100 (white)), a* (−a* = greenness and +a* = redness), and b* (−b* = blueness and +b* = yellowness). Three replications were performed.

#### 2.2.4. Firmness

Considering the method by Zhang et al. [19], the firmness of the samples was measured at two opposite sides by a texture analyzer (TA-XT2i, Stable Micro System Ltd., London, UK) with a 5 mm diameter cylindrical probe. The values were expressed as N. The penetration speed was 0.5 mm/s, and the penetration depth was 5 mm. Ten wolfberries were used for firmness determinations, and three replications were performed.

### 2.3. Antioxidant Content and Activity

#### 2.3.1. Antioxidant Content: Total Phenol and Total Flavonoid Content

The total phenol content was measured by the total phenol test kit (Solarbio, Beijing, China) as described by Wang et al. [20]. Under alkaline conditions, the phenolic substance would reduce the tungsten molybdic acid into a blue compound with a characteristic absorption peak at 760 nm. Briefly, we mixed about 0.1 g of wolfberry fruit powder with 2.5 mL of 60% ethanol solution. Ultrasonic extraction was set at 300 W, 60 °C, and with a 5 s ON and OFF cycle for a total duration of 30 min. The extract was centrifuged at 12,000 rpm at 25 °C for 10 min, and then the supernatant was diluted to 2.5 mL with 60% ethanol solution. Taking 10 μL of the diluted supernatant, we reacted 50 μL of tungsten molybdic acid and then diluted it with distilled water into 200 μL. The absorbance of the extract was measured at 760 nm by the microplate reader (Multiskan MK3, Thermo Lab-systems, Vantaa, Finland). Gallic acid was used as the standard, and the content of total phenol was expressed as mg/g FW (fresh weight). Three replications were performed.

The content of flavonoids was measured by the flavonoid test kit (Solarbio, Beijing, China). Under alkaline nitrite solution conditions, flavonoids and aluminum ions form a red complex with a characteristic absorption peak at 470 nm. About 0.1 g of wolfberry fruit powder was mixed with 1.0 mL of extract solution. Ultrasonic extraction was set at 300 W, 60 °C, and with a 5 s ON and OFF cycle for a total duration of 30 min. The extract was centrifuged at 12,000 rpm at 25 °C for 10 min, and then the supernatant was taken and diluted to 1.0 mL with 60% ethanol solution. The absorbance of the extract was measured at 470 nm by the microplate reader (Multiskan MK3, Thermo Lab-systems, Vantaa, Finland). Rutin was used as the standard, and the content of flavonoids was expressed as mg/g FW. Three replications were performed.

#### 2.3.2. Antioxidant Capacity: DPPH and ABTS In Vitro Activity

The DPPH and ABTS radical scavenging activity were measured by the DPPH and ABTS test kit, respectively (Solarbio, Beijing, China). Fresh fruit samples were put into a 60 °C oven and dried to achieve a constant weight. About 0.05 g of wolfberry fruit powder was mixed with 1.0 mL of the extract solution and then transferred into a water bath operated at 40 °C for 30 min. The mixture was centrifuged at 10,000 rpm at room temperature for 10 min. The absorbance of the supernatant was measured at a wavelength of 515 nm and 405 nm for DPPH and ABTS radical scavenging activity, respectively, by the Microplate reader. Three replications were performed.

#### 2.3.3. Antioxidant Enzyme: SOD and CAT

The superoxide dismutase (SOD) activity was measured by the superoxide dismutase activity test kit (Solarbio, Beijing, China) as reported in previous studies [21]. About 0.1 g of wolfberry fruit powder was mixed with 1.0 mL of extract solution and then centrifuged at 8000 rpm, 4 °C for 10 min. Then, 18 μL of the sample, 45 μL of 100 μmol/L EDTA-Na2 solutions, 2 μL of xanthine oxidase, 35 μL of 130 mmol/L methionine solution, and 90 μL of 750 μmol/L nitroblue tetrazolium solution were added to 96-well plates. The control was left blank with only 18 μL of double-distilled water. The samples were incubated for 30 min at room temperature after mixing and then measured at the wavelength of 560 nm by the Microplate reader. Three replications were performed.

The catalase (CAT) activity was measured by the catalase test kit (Solarbio, Beijing, China). About 0.1 g of wolfberry fruit powder was mixed with 1.0 mL of the extract solution and then centrifuged at 8000 rpm, 4 °C for 10 min. Then, 10 μL of the sample and 190 μL of working solution were added to 96-well plates. The absorbance change of the solution in 1 min was recorded at a wavelength of 240 nm by the Microplate reader. Three replications were performed.

### 2.4. Cell Membrane Permeability, Malondialdehyde (MDA) Content, and Ultrastructure Determination

#### 2.4.1. Cell Membrane Permeability

The cell membrane permeability was measured as mentioned by Carvajal et al. [22]. Fifteen discs in each replicate were taken with an 8 mm diameter hole puncher. Each replicate was rinsed with 20 mL of deionized water and then vibrated for 10 min. After placing samples in a vacuum drier for 10 min, we used a conductivity meter for detecting its initial conductivity (γ1). Later, samples were placed in a boiling (100 °C) water bath for 15 min. After cooling to room temperature, conductivity (γ2) was then detected again by a conductivity meter. Three replications were performed. Relative conductivity was determined by following Equation (3).

(3) $$Relative\;conductivity\;\left(\%\right)=\frac{\gamma 1}{\gamma 2}\times 100\%$$

#### 2.4.2. MDA Content

About 1 g of wolfberry fruit powder was mixed with 5 mL of 100 g/L trichloroacetic acid (TCA) solution, and then centrifuged at 4 °C, 10,000 rpm for 20 min. A total of 2 mL of the supernatant was taken and added with 2 mL of 0.67% thiobarbituric acid (TBA) solution in a boiling water bath for 20 min, then cooled to room temperature in an ice bath and centrifuged again. We measured the absorbance of the supernatant at 450 nm, 532 nm, and 600 nm with a Microplate reader. Three replications were performed. The following equations (Equations (4) and (5)) were used to determine the C_(MDA)_ content and MDA content.

(4) $$c_{\left(\mathrm{MDA}\right)}\frac{\mu \mathrm{mol}}{L}=6.45\times \left({\mathrm{OD}}_{532}-{\mathrm{OD}}_{600}\right)-0.56\times {\;\mathrm{OD}}_{450}\;$$

(5) $$\;\mathrm{MDA}\;\mathrm{content}\;\left(\frac{\mu \mathrm{mol}}{g}\;FW\right)=\frac{c_{\left(\mathrm{MDA}\right)}\times 5}{2\times 1\times 1000}$$

#### 2.4.3. Ultrastructure Determination

According to the method by Wang et al. [23], 1 mm^3^ of the wolfberry tissue was taken from the skin and immersed in 2.5% glutaraldehyde and refrigerated at 4 °C for more than 12 h. After washing for 15 min with 0.1 M sodium phosphate buffer (pH 7.0) three times, the samples were fixed in 1% osmium tetroxide for 2 h. Next, the samples were washed three times with phosphate buffer and then dehydrated in gradient ethanol replacement (30%, 50%, 70%, 80%). After infiltration and embedding, the samples were sliced with the EM UC 7 ultramicrotome (Lycra, Wetzlar, Germany) and stained with uranyl acetate and alkaline lead citrate for 5–10 min. Finally, the Hitachi H-7650 transmission electron microscope (Hitachi High-Technologies Corporation, Tokyo, Japan) was used to observe the slices of wolfberry cells. Three replications were performed.

### 2.5. Cellulose, Proto Pectin, Soluble Pectin

#### 2.5.1. Cellulose Content

The cellulose content was measured by the cellulose test kit (Suobio, Shanghai, China). About 0.3 g of wolfberry fruit powder was mixed with 1.0 mL of 80 % ethanol and heated at 90 °C in a water bath for 20 min and then centrifuged at 6000 rpm, 25 °C for 10 min after cooling to room temperature. The supernatant was discarded, and 1.5 mL of 80% ethanol and acetone (vortex for about 2 min, centrifuge at 6000 rpm, and 25 °C for 10 min) was added successively. To this, 1.0 mL of DMSO (dimethyl sulfoxide) was added and left for 15 h; then, the mixture was centrifuged at 6000 rpm, 25 °C for 10 min; the supernatant was discarded; and the precipitate was dried, in order to obtain cell wall material (CWM).

CWM was placed into the oven at 60 °C and dried until the achievement of a constant weight. In about 5 mg of the dried CWM, we added 0.5 mL of distilled water in an ice water bath; then, we slowly added 0.75 mL of concentrated sulfuric acid. After waiting for 30 min, the mixture was centrifuged at 8000 rpm for 10 min at 4 °C. The supernatant was diluting with distilled water (20 times), and the absorbance was read at 630 nm. Three replications were performed.

#### 2.5.2. Proto Pectin

The proto pectin was measured by the proto pectin test kit (Suobio, Shanghai, China). About 0.05 g of the sample was added to 1 mL of extract solution, and then the mixture was heated in a water bath at 90 °C for 30 min. After cooling, the mixture was centrifuged at 5000 rpm, 25 °C for 10 min. The supernatant was discarded, and 1 mL of ethanol was added to the precipitate and repeated. To this precipitate, 1 mL of the diluted sulfuric acid was added and heated in a water bath at 90 °C for 1 hour. The mixture was again centrifuged at 8000 rpm, 25 °C for 15 min; the supernatant was collected; and the absorbance was read at 530 nm. Three replications were performed.

#### 2.5.3. Soluble Pectin

The soluble pectin was measured by the soluble pectin test kit (Suobio, Shanghai, China). About 3 mg of the dried CWM was added to 1 mL of sodium acetate. The mixture was centrifuged at 8000 rpm at 4 °C for 10 min, the supernatant was collected, and the absorbance was read at 530 nm. Three replications were performed.

### 2.6. Cell Wall Degradation Enzymes

#### 2.6.1. Polygalacturonase (PG)

The polygalacturonase was measured by the polygalacturonase test kit (Suobio, Shanghai, China). About 0.1 g of the sample was mixed with 1 mL of the extract solution and homogenized in the ice bath. The mixture was centrifuged at 16,000 rpm for 10 min at 4 °C, the supernatant was collected, and the absorbance was read at 540 nm. Three replications were performed.

#### 2.6.2. Pectate Lyase (PL)

The pectate lyase content was measured by the pectate lyase test kit (Suobio, Shanghai, China). About 0.1 g of the sample was mixed with 1 mL of the extract solution and homogenized in the ice bath. The mixture was centrifuged at 10,000 rpm for 10 min at 4 °C, the supernatant was collected, and the absorbance was read at 235 nm. Three replications were performed.

#### 2.6.3. Cellulase (CL)

The cellulase content was measured by the cellulase test kit (Suobio, Shanghai, China). About 0.1 g of the sample was mixed with 1 mL of extract solution and homogenized in the ice bath. The mixture was centrifuged at 8000 rpm for 10 min at 4 °C, the supernatant was collected, and the absorbance was read at 550 nm. Three replications were performed.

#### 2.6.4. β-Glucosidase (β-GC)

The β-glucosidase content was measured by the β-glucosidase content test kit (Suobio, Shanghai, China). About 0.1 g of the sample was mixed with 1 mL of extract solution and homogenized in the ice bath. The mixture was centrifuged at 15,000 rpm for 10 min at 4 °C, the supernatant was collected, and the absorbance was read at 400 nm. Three replications were performed.

### 2.7. Statistical Analyses

All data are expressed as ± standard error (SE) from three technical replications and three biological replications. Statistical analysis was performed using SPSS (V23.0) (SPSS Inc., Chicago, IL, USA). The data were analyzed using one-way analysis of variance (ANOVA) to test the significant differences among the treatments. The significant differences were measured at *p* < 0.05.

## 3. Results

### 3.1. Physiological Characteristics

**Figure 1:**
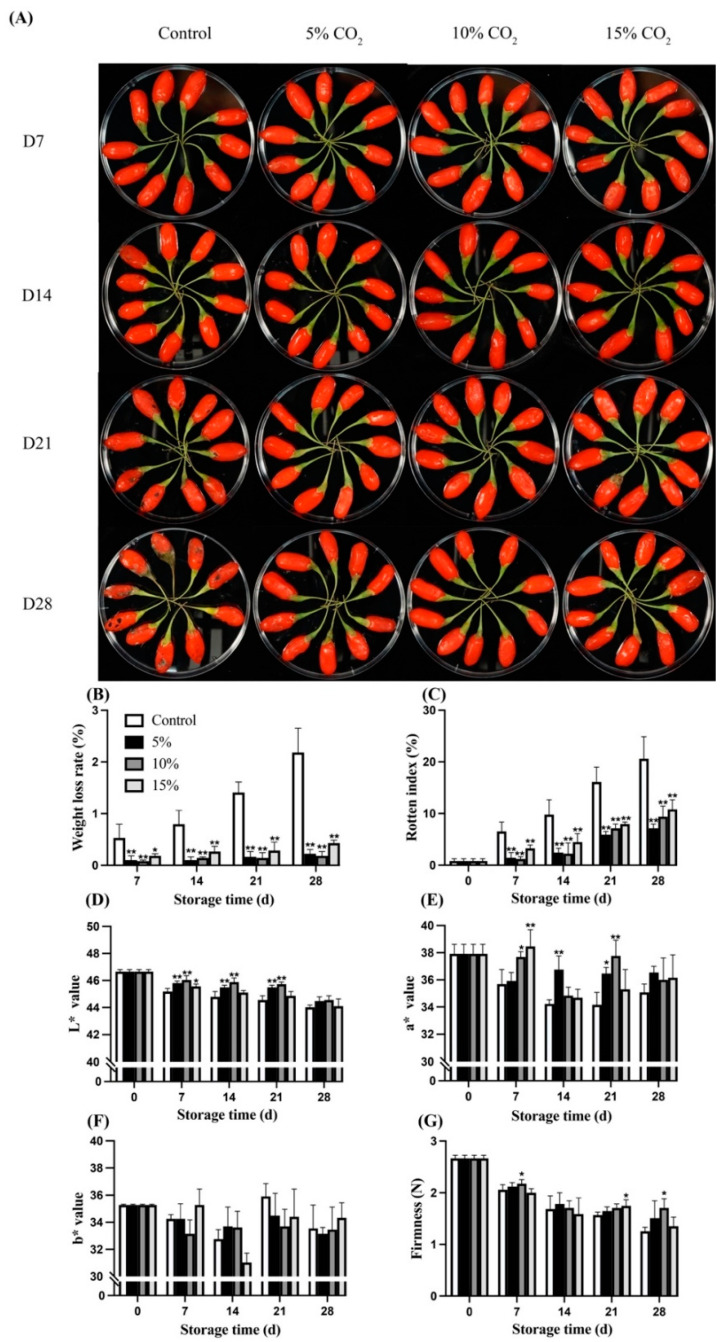
Morphology of wolfberry in response to elevated CO_2_ during storage at 0 ± 0.5 °C for 28 d (**A**), and the effects of elevated CO_2_ on weight loss (**B**), rotten index (**C**), L* value (**D**), a* value (**E**), b* value (**F**), and firmness (**G**) of wolfberry during storage at 0 ± 0.5 °C for 28 d. Significant level * *p* < 0.05; ** *p* < 0.01.

As shown in Figure 1A, the fresh wolfberry fruit can be stored for about 7–10 days under the condition of 0 °C, while the elevated CO_2_ treatment can extend its shelf life to 21 days or more. At the first beginning, the fresh wolfberry fruit had a bright red color, a plump surface, and a bright green carpopodium. On the 7th day, the fruit carpopodium changed from green to yellow, which was slightly dry along with the fruit began to shrink on the 14th day. On the 21st day, the surface of the pericarp appeared to be rotten, and microorganisms proliferated. The 10% CO_2_ treatment group can effectively maintain the bright red color of the fresh wolfberry fruit, the plump surface, and bright green carpopodium during the whole storage period.

The weight loss was observed from the 7th day of storage. Both weight loss and rotten index showed a gradual increase during the storage time (Figure 1B,C). A total of 5% CO_2_ and 10% CO_2_ treatment significantly (*p* < 0.01) inhibited the escalation, with 88.96% and 90.47% lower weight loss, and 64.05% and 53.75% lower rotten index observed at the end of storage time as compared with the control, respectively. As shown in Figure 1D, elevated CO_2_ treatment inhibited the decrease of L* value over the storage time; however, no significant (*p* > 0.05) difference was observed on the 28th day. The 10% CO_2_ treatment showed a more substantial effect on L* value maintenance than 5% CO_2_ and 15% CO_2_ treatment. Samples were getting softened; however, no noticeable difference in the firmness of the wolfberry between the treatments was observed during the entire storage period (Figure 1G).

### 3.2. Antioxidant Activity

**Figure 2:**
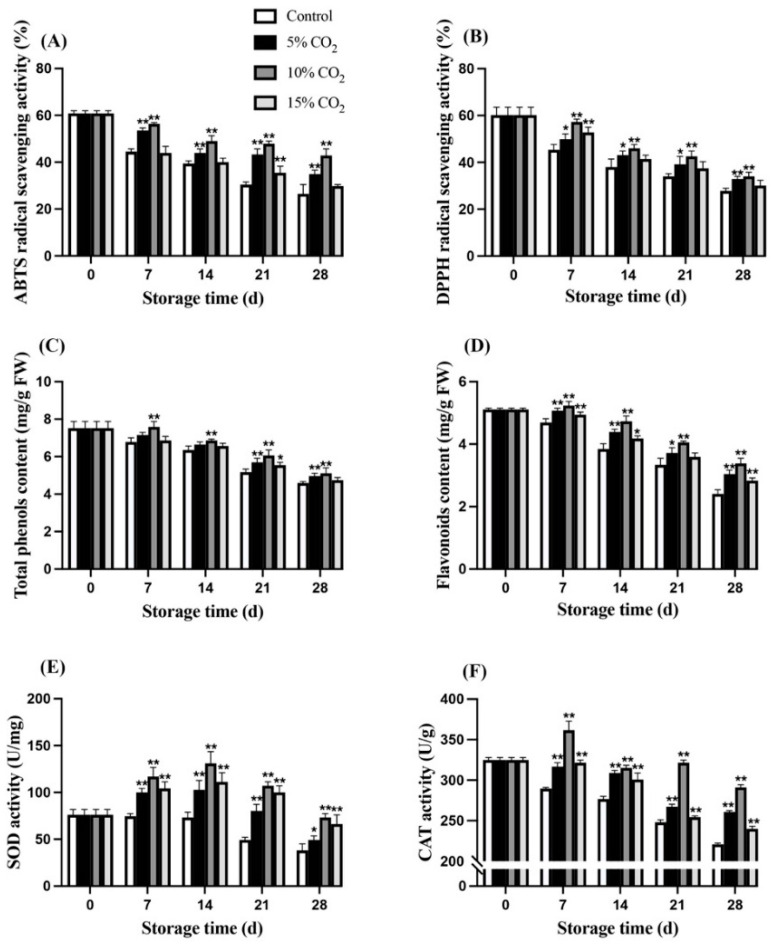
Effects of elevated CO_2_ on ABTS (2,2′-azino-bis (3-ethylbenzothiazoline-6-sulfonic acid)) radical scavenging activity (**A**), DPPH (1,1-diphenyl-1-picrylhydrazil) radical scavenging activity (**B**), total phenol content (**C**), total flavonoid content (**D**), SOD (superoxide dismutase) activity (**E**), and CAT (catalase) activity (**F**) of wolfberry during storage at 0 ± 0.5 °C for 28 d. Significant level * *p* < 0.05; ** *p* < 0.01.

Both the ABTS radical scavenging activity and DPPH radical scavenging activity decreased from the first day of storage (Figure 2A,B), and wolfberries treated with 10% CO_2_ had significantly (*p* < 0.01) higher activity. On the 28th day, the ABTS radical scavenging activities in wolfberries treated with 5% CO_2_ and 10% CO_2_ were 23.15% and 37.18% higher, respectively. In comparison, the DPPH radical scavenging activities in 5% CO_2_ treatment and 10% CO_2_ treatment were 15.65% and 17.95% higher, respectively, than that of the control group. It is worthwhile mentioning that 10% CO_2_ treatment maintained the total phenolic content and total flavonoids in wolfberry at a significantly (*p* < 0.01) higher standard during storage compared with the control (Figure 2C,D). However, wolfberry’s total phenolic content and total flavonoids underwent a marked depletion during storage, losing 30.34% and 33.25% after 28 days of storage in response to 10% CO_2_ treatment, respectively, compared to its initial values. SOD activity in control wolfberries decreased at first, while increasing in the wolfberry treated with 10% CO_2_, peaking at 132.89 U/mg on the 14th day, and then dropping back to the pre-storage level (Figure 2E). Moreover, 10% CO_2_ treatment was able to maintain CAT activity in wolfberry at a significantly (*p* < 0.01) higher level compared with the control group (Figure 2F). Thus, elevated CO_2_ treatment, especially 10% CO_2_ treatment, showed a positive effect on wolfberry total antioxidant activity compared with the control.

### 3.3. Cellular Structure

**Figure 3:**
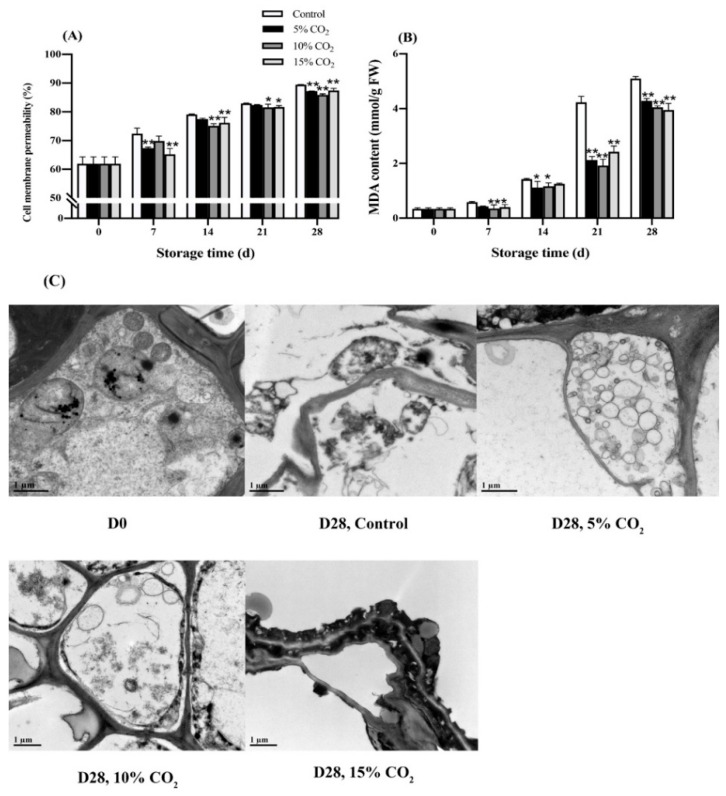
Effects of elevated CO_2_ on cell membrane permeability (**A**), MDA (malondialdehyde) content (**B**), and ultrastructure (**C**) of wolfberry during storage at 0 ± 0.5 °C for 28 d. Significant level * *p* < 0.05; ** *p* < 0.01.

The cell membrane permeability of wolfberry in elevated CO_2_ treatments significantly (*p* < 0.05) increased compared with the control group (Figure 3A). On the 28th day, the cell membrane permeabilities in 5% CO_2_-treated and 10% CO_2_-treated wolfberry were 87.23% and 85.93%, respectively, 2.56% and 4.11% lower than that of the control group, respectively. The MDA content was found increased in both treatments (Figure 3B). There was a sharp increase in MDA content on the 21st day in the control group, reaching 4.23 nmol/g FW, 49.86% and 54.67% higher than 5% CO_2_ treatment and 10% CO_2_ treatment, respectively. On the 28th day, the MDA content in elevated CO_2_ treated wolfberry increased tremendously, rising to a value of 4 nmol/g FW. In addition, the condition of fresh wolfberry fruit under different treatments was also observed by transmission electron microscopy (Figure 3C). On day 0, the cell structure was intact and rarely damaged; however, on the 28th day, the cell wall of the control group was bent and deformed (Figure 3C).

Moreover, the protoplast of the 15% CO_2_-treated cells leaked some contents, resulting in the loss of the original plump state of the plastids, indicating that the cell membrane system was significantly damaged with the senescence of the fresh wolfberry fruit. The cell wall of wolfberry at the 10% CO_2_ treatment was deformed; however, no significant degradation was found, the intercellular layer was clear, and the plasmolysis phenomenon appeared. In the 5% CO_2_ treatment, the inclusions produced by the cytoplasm were close to the cell wall, and the organelle structure appeared to be complete in the treated wolfberry.

### 3.4. Cell Wall Metabolism

**Figure 4:**
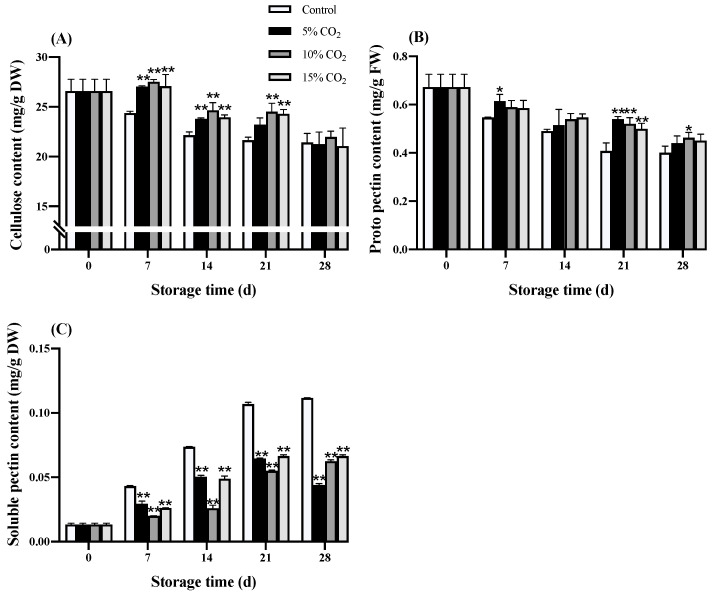
Effects of elevated CO_2_ on cellulose content (**A**), proto pectin content (**B**), and soluble pectin content (**C**) in wolfberry during storage at 0 ± 0.5 °C for 28 d. Significant level * *p* < 0.05; ** *p* < 0.01.

The cellulose and proto pectin contents showed varying degrees of reduction, while and soluble pectin increased gradually during the storage time in both the treatment groups (Figure 4A–C). Wolfberry treated with elevated CO_2_ had higher proto pectin contents; however, the elevation in soluble pectin content was restrained. During the early storage time, elevated CO_2_ treatment in wolfberry had significant (*p* < 0.01) higher cellulose content than the control group, while at day 28, no significant differences were observed. The trend of proto pectin content in both treatment groups was similar, and 10% CO_2_ treatment had 0.46 mg/g FW at day 28, which was 15.36% higher (*p* < 0.05) than the control group. Both elevated CO_2_ treatments had significant (*p* < 0.01) differences in soluble pectin content with the control group. At 5% CO_2_ treatment, wolfberry showed a lower level of soluble pectin content on the 28th day, which was 43.93% lower than the control group.

### 3.5. Cell Wall Metabolism Enzyme Activity

**Figure 5:**
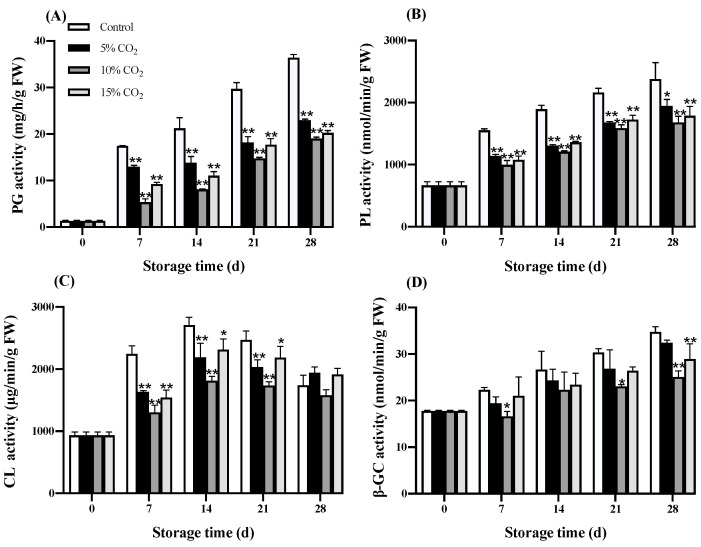
Effects of elevated CO_2_ treatment on PG (polygalacturonase) activity (**A**), PL (pectate lyase) activity (**B**), CL (cellulase) activity (**C**), and β-GC (β-glucosidase) activity (**D**) in wolfberry during storage at 0 ± 0.5 °C for 28 d. Significant level * *p* < 0.05; ** *p* < 0.01.

The PG, PL, CL, and β-GC enzyme activity increased gradually in both treatment groups (Figure 5A–D). Both elevated CO_2_ treatments had significantly (*p* < 0.05) limited the level of PG and PL after the 28th day of storage. After the 21st day of storage, the 5% and 10% CO_2_ treatment showed a significantly (*p* < 0.01) lower CL activity than the control fruit. As for β-GC activity, the elevated CO_2_ effect on wolfberry gradually increased the activity during the entire storage period; however, lower β-GC activity was observed in the elevated CO_2_ wolfberry treatments than in the control. After the 28th day of storage, a significantly (*p* < 0.01) lower β-GC activity was recorded in wolfberry treated with 10% and 15% CO_2_.

## 4. Discussion

MAP acted as an effective method to keep fruits freshness and postharvest quality at optimal gases concentration, limit respiration rate, and prevent senescence from prolonging shelf life [24]. Recently, elevated CO_2_ has been widely researched and applied in fruit preservation. In this study, wolfberry (*Lycium barbarum* L.) fruits were exposed at elevated CO_2_ atmosphere (5 to 15%) to determine the optimal concentration and further investigate the role of antioxidant enzymes and cell wall metabolism in providing better post-harvested fruit quality. Results demonstrated that 10% CO_2_ treatment significantly inhibited the fruit weight loss and decreased L* value, while 5% CO_2_ treatment had better performance in delaying the growth of fruit decay (Figure 1). As expected, elevated CO_2_ treatments showed better physiological characteristics during the storage. It is reported that the jujubes can be stored until day 49 at MAP (14.50 kPa O_2_ and 3.86 kPa CO_2_) combined with cold storage, while the control group showed non-commercial appearance at day 21 [25]. Furthermore, MAP-treated jujubes had significantly (*p* < 0.05) lower weight loss and maintained a higher L* value at the end of the storage period, which was also seen in the present study.

The wolfberry is one of the most antioxidant-rich fruits, generally higher in total phenols and flavonoids contents. However, the antioxidant metabolism changed over time, and the antioxidant activity profile of wolfberry in response to elevated CO_2_ is shown in Figure 2. At 10% CO_2_ treatment, significantly (*p* < 0.01) higher content of total phenols and flavonoids and higher antioxidant enzyme activity in wolfberry were reported. These findings were in accordance with the previous research on half-red strawberries, which also showed higher levels of antioxidants at elevated CO_2_ storage conditions [26]. Moreover, it was confirmed that elevated CO_2_ could increase the activities of SOD and CAT in pear fruit, which may be related to an increased scavenging of reactive oxygen species (ROS) [27]. Furthermore, elevated carbon dioxide may also limit the production of free radicals, which is associated with an increased antioxidant capacity [28].

During the postharvest storage of wolfberry fruit, superoxide anion radicals and hydroxyl radicals are produced in fruits, which can induce membrane lipid peroxidation, leading to an increased cell membrane permeability. However, only 10% CO_2_ treatment exhibited a significant (*p* < 0.05) difference between the control group in firmness, and elevated treatments all significantly (*p* < 0.01) inhibited the increase in cell membrane permeability and MDA content (Figure 3). Inconsistent, Blanch et al. [29] found that strawberries stored at 20% CO_2_ had lower MDA content than the control group on day 3, indicating that elevated CO_2_ may reduce the oxidative stress and delay the lipid peroxidation. For postharvest fruits, the permeability of cell membranes increased with an accelerating rate of electrolyte leakage, which causes an increase in electrical conductivity [30]. Moreover, in agreement with the present study, the controlled atmosphere (5% O_2_ + 5% CO_2_) showed higher levels of membrane integrity than the control during storage periods in pear [31]. In addition, elevated CO_2_ treatment may ameliorate thylakoid injuries and enhance the number of cytoplasmic lipids, which resulted from high storage lipid production due to increased carbon availability [32].

**Figure 6:**
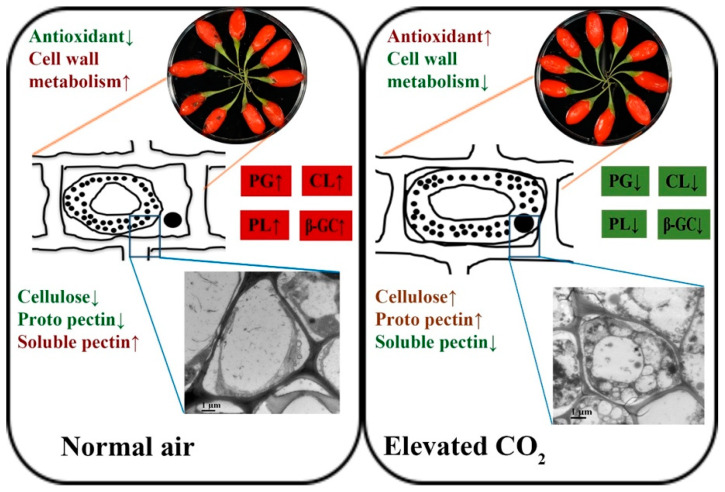
The potential model of elevated CO_2_ on cell wall degradation in wolfberry fruit. The red and green boxes refer to promotion and inhibition effect, respectively.

Softening fruit texture is an important feature during fruit ripening and storage, and it largely influences the consumer preference on selecting fruits. Fruit softening results from changes in cell wall structure and components (mainly cellulose and pectin) and was regulated by a series of cell wall degradation enzymes (Figure 6). It can improve the flavor of the fruit and may also reduce the ability of the fruit to resist external adverse effects and shorten the shelf life [33]. Overall, elevated CO_2_ can effectively prevent the decrease in cellulose content in the early storage period. Moreover, 10% CO_2_ significantly (*p* < 0.05) inhibited the decreased level of proto pectin content, and all elevated CO_2_ treatments significantly (*p* < 0.01) inhibited the increased level of soluble pectin content at the later storage period (Figure 4). Bang et al. [34] found that elevated (30%) CO_2_ treatment could delay the cell wall degradation in strawberries and maintain the integrity of the fruit structure. Siddiqui et al. [35] discovered that apples stored in a controlled atmosphere (3% O_2_ + 3% CO_2_) had a lower decrease in total pectin and hemicellulose contents compared with the control group, while cellulose-bound pectin showed a gradual decline.

Fruit softening is often accompanied by the change of enzyme activity related to cell wall degradation, such as PG, PL, CL, and β-GC. These enzymes degraded pectin polysaccharide components, reduced cell connections, and played different roles in different stages of fruit softening [36]. Both the elevated CO_2_ treatments had a preserving effect on cell wall enzyme activity compared to the control group. It was also evident that at 10% treatment, lower enzymatic activity was recorded on day 28 (Figure 5). PG is the main enzyme that promotes the solubilization of pectin, and the rapid increase in PG activity is remarkably consistent with the cell wall dissolution and the gradual loss of firmness, as seen in many fruits [37]. PL plays a significant role in softening fleshy fruits and is usually a virulence factor leading to plant diseases [38]. Cellulase degrades xyloglucan into cellulose hemicellulose, thereby affecting cell wall structure [39]. CL activity in wolfberry increased on day 14 of the storage period and then decreased rapidly, like the persimmon fruit during the postharvest storage, and may justify by the finding that cellulase is closely related to the early stage of fruit ripening and softening [40]. β-GC can convert cellobiose into glucose, and it has a complicated relationship with the degradation of cellulose [41]. Chang et al. [42] reported that elevated CO_2_ (90 kPa) treatment inhibited the activity of cell wall degrading enzymes, such as PG and β-GC, thereby maintained the firmness of the peach fruits.

## 5. Conclusions

In summary, elevated CO_2_ increased postharvest wolfberry quality; preserving antioxidant activity; and maintaining fruit firmness, structure, color, and overall physical state. Additionally, 10% CO_2_ treatment showed significantly (*p* < 0.01) lower weight loss, rotten index, and higher L* value during the storage period. To illustrate, elevated CO_2_ treatments provided higher free radical scavenging activity, total antioxidant contents, and antioxidant enzyme activities in wolfberry. Moreover, elevated CO_2_ treatments might contribute to retaining fruit firmness, lower cell wall permeability, malondialdehyde content, and intact cell structure. Furthermore, elevated CO_2_ treatments inhibited the growth of water-soluble pectin content and the decrease of proto pectin content, restraining the changes of cell wall-degrading enzymes. In conclusion, for the first time, this study provides a new perspective into the impact of elevated CO_2_ on postharvest wolfberry quality maintenance by increasing the antioxidant enzyme activity and cell wall homeostasis. Further research needs to be conducted on determining the key factors and molecular mechanism of maintaining the fresh fruit quality under MAP conditions.

## Data Availability

Data is contained within the article.
